# Fixed Dystonia in Complex Regional Pain Syndrome: a Descriptive and Computational Modeling Approach

**DOI:** 10.1186/1471-2377-11-53

**Published:** 2011-05-24

**Authors:** Alexander G Munts, Winfred Mugge, Thomas S Meurs, Alfred C Schouten, Johan Marinus, G Lorimer Moseley, Frans CT van der Helm, Jacobus J van Hilten

**Affiliations:** 1 Department of Neurology, Leiden University Medical Center, Leiden, The Netherlands; 2 Department of Biomechanical Engineering, Delft University of Technology, The Netherlands; 3 Prince of Wales Medical Research Institute & University of New South Wales, Sydney, Australia

## Abstract

**Background:**

Complex regional pain syndrome (CRPS) may occur after trauma, usually to one limb, and is characterized by pain and disturbed blood flow, temperature regulation and motor control. Approximately 25% of cases develop fixed dystonia. Involvement of dysfunctional GABAergic interneurons has been suggested, however the mechanisms that underpin fixed dystonia are still unknown. We hypothesized that dystonia could be the result of aberrant proprioceptive reflex strengths of position, velocity or force feedback.

**Methods:**

We systematically characterized the pattern of dystonia in 85 CRPS-patients with dystonia according to the posture held at each joint of the affected limb. We compared the patterns with a neuromuscular computer model simulating aberrations of proprioceptive reflexes. The computer model consists of an antagonistic muscle pair with explicit contributions of the musculotendinous system and reflex pathways originating from muscle spindles and Golgi tendon organs, with time delays reflective of neural latencies. Three scenarios were simulated with the model: (i) increased reflex sensitivity (increased sensitivity of the agonistic and antagonistic reflex loops); (ii) imbalanced reflex sensitivity (increased sensitivity of the agonistic reflex loop); (iii) imbalanced reflex offset (an offset to the reflex output of the agonistic proprioceptors).

**Results:**

For the arm, fixed postures were present in 123 arms of 77 patients. The dominant pattern involved flexion of the fingers (116/123), the wrists (41/123) and elbows (38/123). For the leg, fixed postures were present in 114 legs of 77 patients. The dominant pattern was plantar flexion of the toes (55/114 legs), plantar flexion and inversion of the ankle (73/114) and flexion of the knee (55/114).

Only the computer simulations of imbalanced reflex sensitivity to muscle force from Golgi tendon organs caused patterns that closely resembled the observed patient characteristics. In parallel experiments using robot manipulators we have shown that patients with dystonia were less able to adapt their force feedback strength.

**Conclusions:**

Findings derived from a neuromuscular model suggest that aberrant force feedback regulation from Golgi tendon organs involving an inhibitory interneuron may underpin the typical fixed flexion postures in CRPS patients with dystonia.

## Background

Dystonia is characterized by sustained muscle contractions, frequently causing repetitive twisting movements or abnormal postures [1]. The etiological classification of dystonia discriminates primary (idiopathic) dystonia, in which dystonia is the only clinical sign without any identifiable exogenous cause, from secondary forms in which dystonia is a symptom of an identified neurological condition, such as a focal brain lesion, exposure to drugs or chemicals [2]. Primary dystonia is associated with disturbances of higher order sensory processing including sensory temporal-spatial discrimination, multisensory integration for example between visual and tactile input, and movement representation [3]. These disturbances have been attributed to dysfunction of basal ganglia cortico-striatal-thalamo-cortical motor circuits [1,4-6].

One example of secondary dystonia is the so-called peripherally-induced dystonia which may develop following peripheral tissue or nerve injury [7]. Whereas primary dystonia is typically characterized by prolonged twisting and repetitive movements, peripherally-induced dystonia features abnormal postures (fixed dystonia), the underlying cause of which is unknown [8].

These fixed dystonias occur in about 25% of the patients with complex regional pain syndrome (CRPS) which is usually triggered by a limb injury. CRPS is characterized by persistent pain, autonomic and trophic features [9-11] which reflect the various involvement of mechanisms that underlie inflammation [12-14] and vasomotor dysfunction [15,16]. Fixed dystonia in CRPS may spread to other limbs [17,18] and its prognosis is poor [19,20]. Psychological or personality-based factors have been proposed as predisposing factors for CRPS-related dystonia, but the rationale underpinning this proposal is not clear and evidence is lacking.

One hypothesis underpinning CRPS-related fixed dystonia is that noxious input might interfere with joint and muscle proprioception of the affected body part, which in turn distorts segmental and polysegmental muscle activation during voluntary and reflex movements [21]. Disturbed proprioceptive reflexes have been found in patients with CRPS-related dystonia demonstrated by impaired inhibition of H-reflexes on tendon vibration [22], and disturbed proprioceptive reflexes in posture maintenance experiments using a robot manipulator [23]. We therefore hypothesized that fixed dystonia may result from aberrant proprioceptive reflex strengths of position, velocity or force feedback. Although several independent reports appear to describe similar postures [24-26], a formal categorization of CRPS-related dystonia has not been undertaken. We aimed to fill this critical gap by characterizing the nature of CRPS-related dystonia in 85 patients with CRPS-related dystonia. We subsequently used a neuromuscular model to evaluate whether specific disruptions of the musculotendinous system and reflex loops originating from muscle spindles and Golgi tendon organs could produce fixed dystonia as observed in patients with CRPS.

## Methods

### Clinical evaluation

Eighty-five patients with arm or leg pain who presented to the Neurology department of the Leiden University Medical Center and were diagnosed with CRPS type I and dystonia in one or more extremities, participated (Table 1). CRPS was diagnosed according to the criteria of the International Association for the Study of Pain: patients must have (i) continuing pain, allodynia or hyperalgesia, in which the pain is disproportionate to any inciting event; (ii) evidence at some time of edema, changes in skin blood flow or abnormal sudomotor activity in the region of the pain; and (iii) no condition that would otherwise account for the degree of pain and dysfunction [27]. It is convention to categorize patients as having CRPS type II if a nerve lesion is demonstrable and CRPS type I if a lesion is not demonstrable. This study involved only CRPS type I patients with dystonia of at least one extremity.

**Table 1:** Demographic and clinical characteristics (n = 85).

Characteristic	Value
Mean age, years (SD)	41.3 (13.5)
Sex, n (%)	
Male	5 (5.9)
Female	80 (94.1)
Mean duration of CRPS, years (SD)	11.7 (8.6)
Preceding psychiatric history, n (%)	8 (9.4)
Sensory symptoms, n (%)	
Hyperesthesia, hyperalgesia or allodynia	51 (60.0)
Hypesthesia or hypalgesia	74 (87.1)
Mean duration of dystonia, years (SD)	9.9 (8.6)
Number of dystonic extremities, n (%)	
1	8 (9.4)
2	26 (30.6)
3	26 (30.6)
4	25 (29.4)
Severity dystonia most affected extremity, n (%)	
slight	10 (11.8)
mild	35 (41.2)
moderate	21 (24.7)
severe	19 (22.3)

SD = standard deviation.

None of the patients had a history of birth trauma or abnormal development. Other causes of dystonia had been excluded using appropriate blood and imaging studies (computed tomography, magnetic resonance imaging) of the spinal cord and brain.

The research was carried out in compliance with the Declaration of Helsinki and all patients provided informed consent before they were filmed while sitting or standing so that sufficient footage of each limb was obtained to characterize its posture. Footage of CRPS patients who exhibited fixed dystonia at rest on clinical examination between 1994 and 2007 was examined by one investigator (TSM). The severity of dystonia in the affected extremities was evaluated using the severity factor of the Burke-Fahn-Marsden scale (slight, mild, moderate or severe) [28]. Patterns of fixed posture were evaluated in four joints of the arms (fingers, wrist, elbow and shoulder) and legs (toes, ankle, knee and hip). Medical records were evaluated to verify that the posture observed in the footage was consistent with clinical presentation.

### Neuromuscular model simulation of fixed dystonia

The neuromuscular model used to simulate dystonia consists of two antagonistic muscles attached to hand inertia, with Hill-type activation and contraction dynamics [29] based on the Winters and Stark muscle model [30]. Two parameter sets for the wrist and shoulder muscles were adopted from Winters and Stark [30]. The model contains explicit contributions of the musculotendinous system and reflex pathways originating from muscle spindles and GTO with subsequent time delays to represent neural latencies. In the model, three reflex pathways are included that excite the contractile element of the muscle: (i) velocity-dependent pathways initiated by activation of type Ia afferents from the muscle spindles; (ii) position-dependent pathways initiated by activation of type II afferents from the muscle spindles; and (iii) force-dependent pathways initiated by activation of type Ib afferents (GTO) (Table 2). The model enables determination of the contribution of each of these pathways to muscle activity in the arm.

**Table 2:** Proprioceptive feedback pathways in humans

Feedback Pathway	Proprioceptive Sensory Organ	Afferent Nerve Type	Physical Measure	Sensitive to Muscle Shortening or Lengthening
Position	Muscle spindle	Mainly II	Muscle stretch	Only lengthening (unidirectional)
Velocity	Muscle spindle	Mainly Ia	Muscle stretch velocity	Only lengthening (unidirectional)
Force	Golgi tendon organ	Ib	Muscle force	Both (bidirectional)

**Figure 1:**
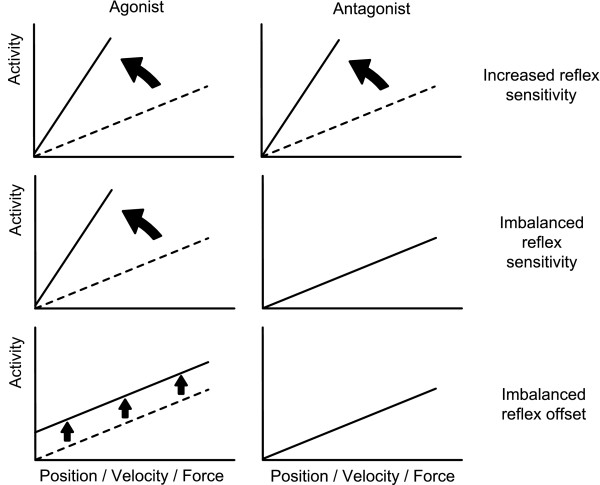
**Schematic representation of the aberrant reflex scenarios tested with the neuromuscular model**. Increased reflex sensitivity, i.e. increased sensitivity of both the agonistic and antagonistic reflex loops; imbalanced reflex sensitivity, i.e. increased sensitivity of only the agonistic reflex loop; and imbalanced reflex offset, i.e. an offset to the reflex output in only the agonistic proprioceptors.

There was one reference scenario with normal reflexes and three scenarios with abnormal reflexes (Figure 1): (i) increased reflex sensitivity, increased sensitivity of both the agonistic and antagonistic reflex loops, i.e. 'hyperreflexia'; (ii) imbalanced reflex sensitivity, increased sensitivity of only the agonistic reflex loop; and (iii) imbalanced reflex offset, an offset to the reflex output in only the agonistic proprioceptors. Each scenario was applied to each of the three reflex pathways to produce nine aberrant conditions.

Although under normal conditions, reflex strength adapts during external force and voluntary movement [31] and reflexes are suppressed to enable unimpeded voluntary movements, we excluded reflex adaptation so that both external forces and voluntary movements elicited reflexes. Each simulation had the same set-up: five seconds of continuous external force were followed by five seconds of rest, and then, five seconds of continuous voluntary contraction followed by again five seconds of rest.

Figure 1 shows that the model reflex strengths were set up as (over) excitatory to agree with neurophysiologic studies in patients with CRPS that have demonstrated reduced central inhibition - so-called 'disinhibition' [18,22,32,33]. Behavior of the resultant model in each of the aberrant reflex scenarios was scored (0-5) according to the following characteristics, which are typical of CRPS-related dystonia [18,34]: (i) abnormal posture; (ii) sustained contraction; (iii) increased stiffness; (iv) worsening with activity; and (v) loss of voluntary control.

## Results

Eighty-five patients with CRPS and fixed dystonia (80 female) participated. Mean (standard deviation) age was 41 (13) with a range from 16 to 69 years. Mean duration of CRPS was 11.7 (8.6) years, mean duration of dystonia was 9.9 (8.6) years and median number of dystonic extremities was 3.

### Arms

**Figure 2:**
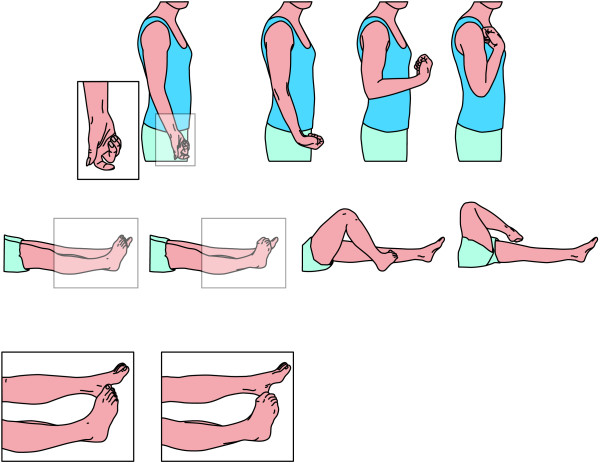
**Dystonic postures in CRPS**. Most common postures in arm and leg in CRPS-related dystonia arranged to the severity from left to right.

Fixed dystonia was apparent in 123 arms of 77 patients (both arms were affected in 46/77 patients). The dominant pattern of fixed dystonia was flexion. This fixed flexion dystonia was more often present in distal joints than in proximal joints, affecting mostly the fingers (116/123) but also the wrist (41/123), and elbow (38/123). Shoulder adduction was observed in 12/123 arms (Figure 2). One or more of these joint postures were found in 118 arms (Table 3A). Other fixed dystonias were observed, although they were much less common. Extension of the fingers was observed in 5/123 arms, extension of the wrist in 1/123, and pronation of the elbow in 3/123.

**Table 3:** Combinations of most common arm (A) and leg postures (B) in patients with CRPS related dystonia

A				
Number of Arms	Flexion Fingers	Flexion Wrist	Flexion Elbow	Adduction Shoulder
60	X			
20	X	X	X	
15	X	X		
9	X		X	
4	X		X	X
4	X	X	X	X
3	X			X
1	X	X		X
1		X		
1			X	

118	116	41	38	12

				

**B**				
**Number of Legs**	**Plantar Flexion Toes**	**Plantar Flexion or Inversion Ankle**	**Flexion Knee**	**Endorotation Hip**

42		X		
24	X	X		
17	X	X	X	
6	X			
6		X	X	
6		X		X
4			X	
3	X	X		X
3	X		X	
2	X	X	X	X

113	55	100	32	11

Most common arm postures were present in 74 patients and most common leg postures in 77 patients. Column totals are presented in the bottom row. Note that for clarity reasons, other postures that occurred with or without these most common postures are not shown.

The extent and nature of fixed dystonia did not vary between left and right arms (p = 0.95, Fisher's exact test). The 60 arms in which at least two segments were involved showed a gradual spread of dystonia from distal to more proximal regions of the limb.

### Legs

Fixed dystonia was present in 114 legs of 77 patients. Only the right leg was involved in 21/77, only the left in 19/77 and both legs in 37/77. Also in the legs, fixed dystonia was more often seen in distal than in proximal joints. The most common postures were plantar flexion and inversion of the ankle (73/114 legs); plantar flexion without inversion (11/114 legs), and inversion without plantar flexion (16/114 legs). Other common postures were plantar flexion in the toes (55/114 legs), flexion of the knee (32/114), and internal rotation in the hip (11/114) (Figure 2). One or more of these postures were observed in 113 legs (Table 3B).

Other postures were rarely observed: dorsal flexion of the ankle (2/114 legs), eversion (2/114), dorsal flexion and inversion (1/114), and plantar flexion and eversion (2/114); dorsiflexion of the toes (4/114), and knee extension (7/114).

As for the arm, there was no difference in the number or nature of fixed dystonias between the left and right legs (p = 0.90, Fisher's exact test). The 73 legs in which at least two segments were involved showed also a gradual spread of dystonia from distal to more proximal regions of the limb.

### Simulating CRPS-related dystonia by modeling aberrant proprioceptive reflexes

**Figure 3:**
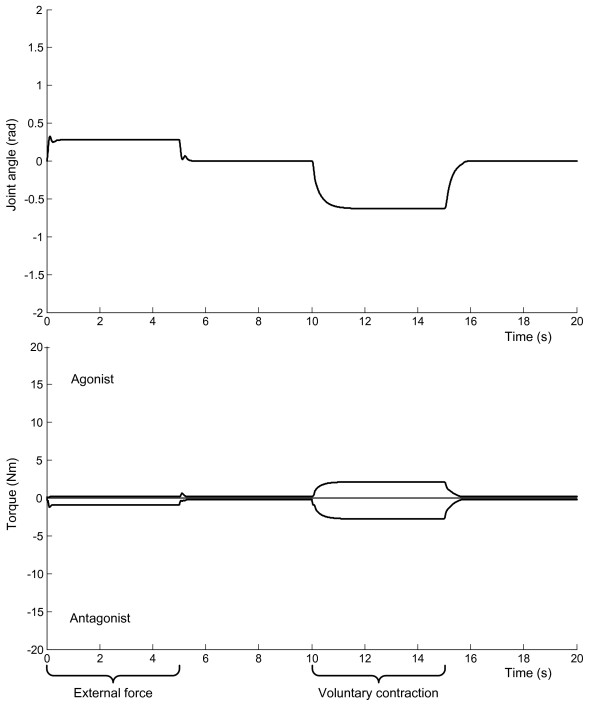
**Model simulation results with normal reflexes**. Joint angle (top panel) and muscle torques (bottom panel) at the wrist in response to external force (0-5 s) and voluntary contraction (10-15 s) with normal reflexes. In periods of rest (5-10 and 15-20 s) the muscle contractions subside and the hand returns to its neutral position.

**Figure 4:**
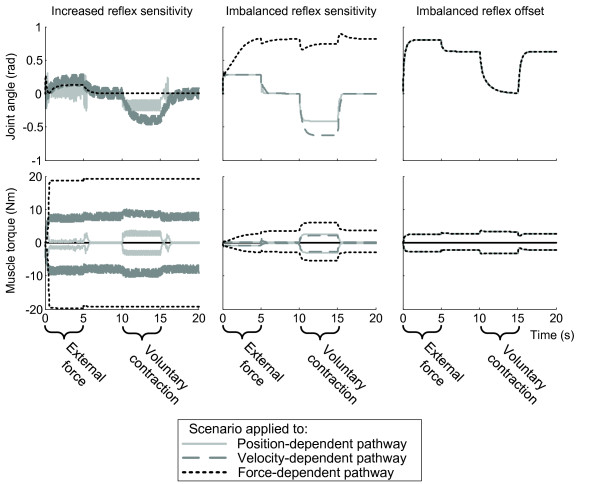
**Model simulation results with the three aberrant reflex scenarios applied to the three reflex pathways**. Joint angles (top panels) and muscle torques (bottom panels) at the wrist in response to external force (0-5 s) and voluntary contraction (10-15 s) with the three aberrant reflex scenarios applied to the three reflex pathways. Left panels show the increased reflex sensitivity scenario, middle panels show the imbalanced reflex sensitivity scenario, and right panels show the imbalanced reflex offset scenario. The three traces within a panel represent the results of the scenario applied to the velocity-, position- and force-dependent pathways.

We used a neuromuscular model to simulate the dominant postures observed in patients. This model incorporates the interaction between mechanical properties of the limb and spinal proprioceptive reflexes. Figure 3 shows the movement and muscle torques of the wrist in the reference condition with (arbitrary) normal reflexes. Figure 4 shows the aberrant reflex scenarios, i.e. (i) increased reflex sensitivity; (ii) imbalanced reflex sensitivity; and (iii) imbalanced reflex offset, which were successively applied to proprioceptive feedback pathways originating from muscle spindles and Golgi tendon organs (Table 2).

**Figure 5:**
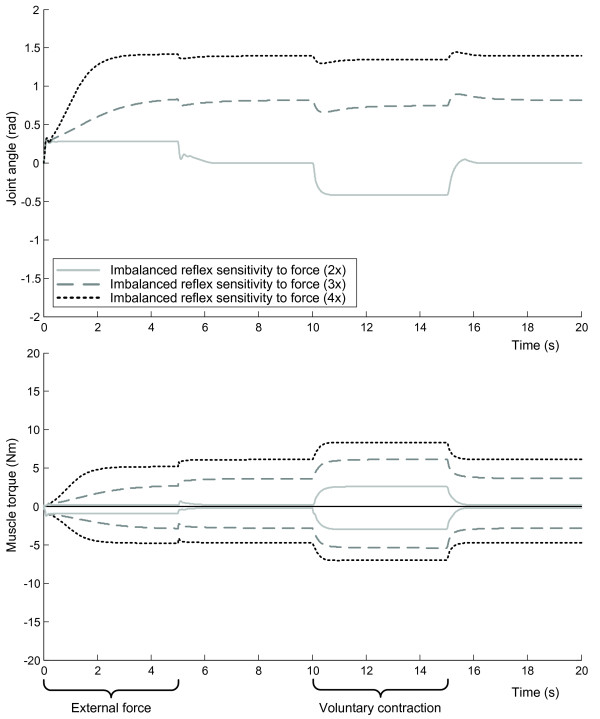
**Model simulation results with several degrees of imbalanced reflex sensitivity to muscle force**. Joint angles (top panel) and muscle torques (bottom panel) at the wrist in response to external force (0-5 s) and voluntary contraction (15-20 s) with several degrees of imbalanced reflex sensitivities to muscle force. The motor behavior resembles fixed dystonia.

The increased reflex sensitivity scenario (i.e. 'hyperreflexia') resulted in motor dysfunction, varying from rigidity (in case of increased reflex sensitivity to force) to fast oscillatory movements (in case of increased reflex sensitivity to velocity or position), but did not cause an abnormal posture (Figure 4). The imbalanced reflex offset scenario resulted in abnormal postures, however, without other characteristics of fixed dystonia such as sustained contraction, increased stiffness and loss of voluntary control (Figure 4). For both the wrist and the shoulder parameter sets the simulation of the imbalanced reflex sensitivity to muscle force was the only condition that resulted in behavior that closely resembled all clinical characteristics of fixed dystonia (Figure 4). The upper plot in Figure 5 shows the deviant joint angles (abnormal posture) that resulted from imbalance of muscle force feedback. The degree of imbalance determined the severity of the deviation, since the force imbalance is counteracted by force contributions from muscle stretch in the antagonist. The increased co-contraction is evident from the high muscle torques in agonist and antagonist in Figure 5. After attaining the abnormal posture, the joint movement induced by external force and voluntary contraction is smaller due to the co-contraction and excitatory force feedback.

## Discussion

By systematically evaluating 123 affected arms and 114 affected legs, of 85 patients with CRPS-related dystonia, we identified a dominant pattern of fixed dystonia that would be predicted on the basis of proprioceptive disruption as an underlying cause. Symptoms are more often present in distal than in proximal joints, and more in flexor than in extensor muscles. In fact, Table 3A shows that proximal joint involvement was always found to be accompanied by more distal dystonia. From the 12 arms with an affected shoulder joint all had affected fingers. In legs the same relation was found between the hip and ankle, from the 11 legs with an affected hip joint all had affected ankles. In contrast, distal dystonias without involvement of proximal joints occurred often: only the fingers were affected in 60 out of 118 arms and only the ankle in 42 out of 113 legs.

The conspicuous involvement of flexor muscles in fixed dystonia in CRPS has been attributed to disinhibition of spinal circuitry involved in mediating nociceptive withdrawal reflexes (NWRs) [18]. The character of the NWR represents the most appropriate movement for a withdrawal of the stimulated area from an offending stimulus [35]. If disinhibition of NWRs played a role in our findings, one would generally expect a stereotypical pattern of multi-segmental muscle involvement. However, only in the most severely affected cases did we encounter such multi-segmental patterns. In the majority of cases there was a selective distal muscle involvement which thus raises the need for an alternative explanation for fixed dystonia.

Indeed, findings from different lines of research are showing that multiple factors may contribute to the development of fixed dystonia in CRPS. First, there are significant sympathetic effects on the sensorimotor system; sympathetic fibers can be traced to bag chain intrafusal fibers as well as ventral muscle efferents [36,37]. Intrafusal fibers of the muscle spindle receive a large diameter primary annulospiral afferent innervation of group Ia fibers [38]. Sympathetic fibers may thus influence skeletal muscle contraction, neuromuscular transmission, anterior horn cell depolarization, and spinal cord reflexes [39-43]. Second, there is emerging evidence implicating peripheral and central N-methyl-D-aspartic (NMDA)-receptor activation in the etiology of motor dysfunction in CRPS [44]. The NMDA receptor antagonist ketamine improved motor impairments in patients with CRPS [44,45]. Third, motor impairments may simply relate to adaptive changes within the central nervous system of patients with CRPS [32]. Finally, flexor motor neurons and associated interneurons which mediate depolarization of primary afferent fibers, receive more sensory input than their extensor counterparts. The release of GABA by spinal interneurons produces primary afferent depolarization and reduces transmitter release (presynaptic inhibition), which in turn modulates reflex gains. The synaptic effectiveness of Ib-afferent feedback ending in the spinal cord of vertebrates can thus be modulated by means of specific sets of GABAergic interneurons [46]. Dysfunction of GABAergic interneurons, which is a key component of central sensitization, has been shown to compromise the specificity of afferent processing [47]. Several studies have found evidence of disinhibition along the neuraxis in CRPS patients with and without dystonia [22,33]. Dystonia in CRPS patients responds to the γ-aminobutyric acid receptor (GABA) B agonist, baclofen, which enhances spinal GABAergic inhibition [48,49] but not to the administration of the inhibitory neurotransmitter glycine [50]. These findings may thus suggest that GABAergic circuits are involved in fixed dystonia in CRPS.

Since separate sets of GABAergic interneurons allow for selective control of muscle length and muscle tension, the predominant flexor postures in dystonia of CRPS may implicate imbalanced control of functionally coupled muscles [51].

We tested three types of aberrant reflex patterns using a neuromuscular model that captures the interaction between proprioceptive reflexes, the mechanical properties of the limb and its load. The aberrant reflex pattern that most closely mimicked the fixed dystonia in patients with CRPS was imbalanced reflex sensitivity to muscle force feedback. The severity of the abnormal posture varied according to the degree of imbalance. In contrast, increased and imbalanced reflex sensitivity to position and velocity feedback only caused oscillatory motions, which likely can be explained by consecutive reflexive contractions leading to decreased stretch in one of the antagonistic muscles, but increased stretch in the other. Increased reflex sensitivity to muscle force caused behavior that exhibited all the characteristics of dystonia, except for the abnormal posture due to the balanced force feedback. Our findings therefore implicate possible involvement of GTO-afferent input. GTO functions as the sensor in the feedback system that regulates muscle force and accurately signals active contractile force [52-54]. Stretch of the tendon, which is proportional to the force in the muscle during active contraction, activates GTO and thereby increases type Ib-afferent input onto inhibitory interneurons subserving primary afferent depolarization. These in turn inhibit α-motor neurons that supply the muscle from which they arise [55]. Finally, since time delays destabilize feedback systems and the delay is greater distally than proximally, disruption of GTO feedback would most likely be associated with fixed dystonias that arise distally and then progress proximally. Hence, central sensitization may impair the processing of GTO-afferent input and thus contribute to the development of fixed dystonia.

Alternatively, peripheral factors that influence the torque at the joint, such as changes of the contractile properties of the muscles, may introduce imbalances in force feedback independent of reflex settings. In fact, differences in agonistic and antagonistic muscle strength and moment arms may already introduce imbalances and possibly only become symptomatic with disturbed feedback control. Speculatively adequate control of reflexes mat be required to actively balance feedback control. The most common ankle postures in our patients were plantar flexion or inversion, and indeed, the contributing muscles have greater strength compared to their antagonists. It may also explain the greater diversity in shoulder and hip postures, because the proportional strength of the contributing muscles is more variable between subjects and postures.

## Conclusions

Findings derived from a neuromuscular model suggest that aberrant force feedback regulation from Golgi tendon organs involving an inhibitory interneuron may underpin the typical fixed flexion postures in CRPS patients with dystonia.

## Competing interests

The authors declare that they have no competing interests.

## Authors' contributions

Conceived and designed the experiments: AGM WM TSM ACS JM GLM FCTH JJH. Performed the experiments: AGM WM TSM. Clinical patient characterization: AGM TSM. Model development and simulation: WM ACS. Analyzed the data: AGM WM TSM. Wrote the paper: AGM WM TSM ACS JM GLM FCTH JJH. All authors read and approved the manuscript.

## Pre-publication history

The pre-publication history for this paper can be accessed here:

http://www.biomedcentral.com/1471-2377/11/53/prepub

## References

[B1] Fahn S Bressman SB Marsden CD Classification of dystonia Adv Neurol 1998 78 1 10 9750897

[B2] Albanese A Asmus F Bhatia KP Elia AE Elibol B EFNS guidelines on diagnosis and treatment of primary dystonias European Journal of Neurology 2011 18 5 18 10.1111/j.1468-1331.2010.03042.x 20482602

[B3] Tinazzi M Fiorio M Fiaschi A Rothwell JC Bhatia KP Sensory functions in dystonia: insights from behavioral studies Mov Disord 2009 24 1427 1436 10.1002/mds.22490 19306289

[B4] Berardelli A Rothwell JC Hallett M Thompson PD Manfredi M The pathophysiology of primary dystonia Brain 1998 121 1195 1212 10.1093/brain/121.7.1195 9679773

[B5] Bressman SB Dystonia Curr Opin Neurol 1998 11 363 372 9725083 10.1097/00019052-199808000-00013

[B6] Hallett M Physiology of dystonia Adv Neurol 1998 78 11 18 9750898

[B7] Jankovic J Peripherally induced movement disorders Neurologic Clinics 2009 27 821 32 10.1016/j.ncl.2009.04.005 19555833

[B8] van Hilten JJ Geraedts EJ Marinus J Peripheral trauma and movement disorders Parkinsonism Relat Disord 2007 13 Suppl 3 S395 S399 18267271 10.1016/S1353-8020(08)70037-3

[B9] Allen G Galer BS Schwartz L Epidemiology of complex regional pain syndrome: a retrospective chart review of 134 patients Pain 1999 80 539 544 10.1016/S0304-3959(98)00246-2 10342415

[B10] Janig W Baron R Complex regional pain syndrome: mystery explained? Lancet Neurol 2003 2 687 697 10.1016/S1474-4422(03)00557-X 14572737

[B11] Veldman PH Reynen HM Arntz IE Goris RJ Signs and symptoms of reflex sympathetic dystrophy: prospective study of 829 patients Lancet 1993 342 1012 1016 10.1016/0140-6736(93)92877-V 8105263

[B12] Birklein F Schmelz M Schifter S Weber M The important role of neuropeptides in complex regional pain syndrome Neurology 2001 57 2179 2184 11756594 10.1212/wnl.57.12.2179

[B13] Huygen FJ de Bruijn AG De Bruin MT Groeneweg JG Klein J Evidence for local inflammation in complex regional pain syndrome type 1 Mediators Inflamm 2002 11 47 51 10.1080/09629350210307 11930962 PMC1781643

[B14] Leis S Weber M Isselmann A Schmelz M Birklein F Substance-P-induced protein extravasation is bilaterally increased in complex regional pain syndrome Exp Neurol 2003 183 197 204 10.1016/S0014-4886(03)00163-8 12957502

[B15] Niehof SP Huygen FJ van der Weerd RW Westra M Zijlstra FJ Thermography imaging during static and controlled thermoregulation in complex regional pain syndrome type 1: diagnostic value and involvement of the central sympathetic system Biomed Eng Online 2006 5 30 10.1186/1475-925X-5-30 16689997 PMC1479347

[B16] Wasner G Schattschneider J Heckmann K Maier C Baron R Vascular abnormalities in reflex sympathetic dystrophy (CRPS I): mechanisms and diagnostic value Brain 2001 124 587 599 10.1093/brain/124.3.587 11222458

[B17] Schott GD Peripherally-triggered CRPS and dystonia Pain 2007 130 203 207 10.1016/j.pain.2007.04.013 17512665

[B18] van Hilten JJ Blumberg H Schwartzman RJ Wilson P, Stanton-Hicks M, Harden RN Factor IV: Movement Disorders and Dystrophy--Pathophysiology and Measurement CRPS: Current Diagnosis and Therapy, Progress in Pain Research and Management 2005 Seattle: IASP Press 119 137

[B19] Ibrahim NM Martino D van de Warrenburg BP Quinn NP Bhatia KP The prognosis of fixed dystonia: a follow-up study Parkinsonism Relat Disord 2009 15 592 597 10.1016/j.parkreldis.2009.02.010 19442561

[B20] Schrag A Trimble M Quinn N Bhatia K The syndrome of fixed dystonia: an evaluation of 103 patients Brain 2004 127 2360 2372 10.1093/brain/awh262 15342362

[B21] McCrea DA Cordo P, Harnad S Can sense be made of spinal interneuron circuits? Movement control 1994 Cambridge: Cambridge University Press 31 41

[B22] van de Beek WJ Vein A Hilgevoord AA van Dijk JG van Hilten BJ Neurophysiologic aspects of patients with generalized or multifocal tonic dystonia of reflex sympathetic dystrophy J Clin Neurophysiol 2002 19 77 83 10.1097/00004691-200201000-00011 11896357

[B23] Schouten AC van de Beek WJ van Hilten JJ Van der Helm FC Proprioceptive reflexes in patients with reflex sympathetic dystrophy Exp Brain Res 2003 151 1 8 10.1007/s00221-003-1420-x 12743675

[B24] Marsden CD Obeso JA Traub MM Rothwell JC Kranz H Muscle spasms associated with Sudeck's atrophy after injury Br Med J (Clin Res Ed) 1984 288 173 176 10.1136/bmj.288.6412.173 PMC1444500 6198018

[B25] Schwartzman RJ Kerrigan J The movement disorder of reflex sympathetic dystrophy Neurology 1990 40 57 61 2296383 10.1212/wnl.40.1.57

[B26] van Rijn MA Marinus J Putter H van Hilten JJ Onset and progression of dystonia in complex regional pain syndrome Pain 2007 130 287 293 10.1016/j.pain.2007.03.027 17499924

[B27] Merskey H Bogduk N Merskey H, Bogduk N Relatively generalized syndromes Classification of chronic pain. Description of chronic pain syndromes and definitions of pain terms 1994 Seattle: IASP Press 40 43

[B28] Burke RE Fahn S Marsden CD Bressman SB Moskowitz C Validity and reliability of a rating scale for the primary torsion dystonias Neurology 1985 35 73 77 3966004 10.1212/wnl.35.1.73

[B29] Stroeve S Impedance characteristics of a neuromusculoskeletal model of the human arm I. Posture control Biol Cybern 1999 81 475 494 10.1007/s004220050577 10592022

[B30] Winters JM Stark L Analysis of fundamental human movement patterns through the use of in-depth antagonistic muscle models IEEE Trans Biomed Eng 1985 32 826 839 4054926 10.1109/TBME.1985.325498

[B31] Johnson MT Kipnis AN Lee MC Ebner TJ Independent control of reflex and volitional EMG modulation during sinusoidal pursuit tracking in humans Exp Brain Res 1993 96 347 362 8270027 10.1007/BF00227114

[B32] Maihofner C Baron R DeCol R Binder A Birklein F The motor system shows adaptive changes in complex regional pain syndrome Brain 2007 130 2671 2687 10.1093/brain/awm131 17575278

[B33] Schwenkreis P Janssen F Rommel O Pleger B Volker B Bilateral motor cortex disinhibition in complex regional pain syndrome (CRPS) type I of the hand Neurology 2003 61 515 519 12939426 10.1212/wnl.61.4.515

[B34] van Hilten JJ van de Beek WJ Vein AA van Dijk JG Middelkoop HA Clinical aspects of multifocal or generalized tonic dystonia in reflex sympathetic dystrophy Neurology 2001 56 1762 1765 11425951 10.1212/wnl.56.12.1762

[B35] Pierrot-Deseilligny E Burke D The circuitry of the human spinal cord: its role in motor control and movement disorders 2005 New York: Cambridge University Press

[B36] Santini M Ibata Y The fine structure of thin unmyelinated axons within muscle spindles Brain Res 1971 33 289 302 10.1016/0006-8993(71)90104-1 4332501

[B37] Barker D Saito M Autonomic innervation of receptors and muscle fibres in cat skeletal muscle Proc R Soc Lond B Biol Sci 1981 212 317 32 10.1098/rspb.1981.0042 6115396

[B38] Grünewald RA Yoneda Y Shipman JM Sagar HJ Idiopathic focal dystonia: a disorder of muscle spindle afferent processing? Brain 1997 120 2179 85 10.1093/brain/120.12.2179 9448573

[B39] Bowman WC Goldberg AA Raper C A comparison between the effects of a tetanus and the effects of sympathomimetic amines on fast- and slow-contracting mammalian muscles Br J Pharmacol Chemother 1962 19 464 84 14014654 10.1111/j.1476-5381.1962.tb01451.x PMC1482211

[B40] Kuba K Effects of catecholamines on the neuromuscular junction in the rat diaphragm J Physiol 1970 211 551 70 5501051 10.1113/jphysiol.1970.sp009293 PMC1396086

[B41] Francini F Peruzzi P Staderini G Effects of sympathetic lumbar trunk stimulation on the myotatic reflex activity of the quadriceps muscle in decerebrate cat Boll Soc Ital Biol Sper 1978 54 1353 6 743436

[B42] Snider RM Gerald MC Studies on the mechanism of (+)-amphetamine enhancement of neuromuscular transmission: muscle contraction, electrophysiological and biochemical results J Pharmacol Exp Ther 1982 221 14 21 6121051

[B43] Grassi C Filippi GM Passatore M Tension development in lumbrical muscles and concomitant increase of activity in A-alpha and A-beta afferents during sympathetic stimulation in the cat Brain Res 1987 435 15 23 10.1016/0006-8993(87)91581-2 3427449

[B44] Kiefer RT Rohr P Ploppa A Dieterich HJ Grothusen J Koffler S Altemeyer KH Unertl K Schwartzman RJ Efficacy of ketamine in anesthetic dosage for the treatment of refractory complex regional pain syndrome: an open-label phase II study Pain Med 2008 9 1173 201 10.1111/j.1526-4637.2007.00402.x 18266808

[B45] Schwartzman RJ Alexander GM Grothusen JR Paylor T Reichenberger E Perreault M Outpatient intravenous ketamine for the treatment of complex regional pain syndrome: a double-blind placebo controlled study Pain 2009 147 1-3 107 15 10.1016/j.pain.2009.08.015 19783371

[B46] Rudomin P Presynaptic selection of afferent inflow in the spinal cord J Physiol Paris 1999 93 329 347 10.1016/S0928-4257(00)80061-3 10574122

[B47] Buesa I Ortiz V Aguilera L Torre F Zimmermann M Disinhibition of spinal responses to primary afferent input by antagonism at GABA receptors in urethane-anaesthetised rats is dependent on NMDA and metabotropic glutamate receptors Neuropharmacology 2006 50 585 594 10.1016/j.neuropharm.2005.11.005 16412481

[B48] Saito K Konishi S Otsuka M Antagonism between Lioresal and substance P in rat spinal cord Brain Research 1975 97 177 180 10.1016/0006-8993(75)90928-2 1175037

[B49] van Hilten BJ van de Beek WJ Hoff JI Voormolen JH Delhaas EM Intrathecal baclofen for the treatment of dystonia in patients with reflex sympathetic dystrophy New England Journal of Medicine 2000 343 625 630 10.1056/NEJM200008313430905 10965009

[B50] Munts AG van der Plas AA Voormolen JH Marinus J Teepe-Twiss IM Intrathecal glycine for pain and dystonia in complex regional pain syndrome Pain 2009 146 199 204 10.1016/j.pain.2009.07.030 19683392

[B51] Rudomin P Presynaptic inhibition of muscle spindle and tendon organ afferents in the mammalian spinal cord Trends Neurosciences 1990 13 499 505 10.1016/0166-2236(90)90084-N 1703681

[B52] Crago PE Houk JC Rymer WZ Sampling of total muscle force by tendon organs J Neurophysiol 1982 47 1069 1083 7108572 10.1152/jn.1982.47.6.1069

[B53] Houk J Henneman E Responses of Golgi tendon organs to active contractions of the soleus muscle of the cat J Neurophysiol 1967 30 466 481 6037588 10.1152/jn.1967.30.3.466

[B54] Jami L Golgi tendon organs in mammalian skeletal muscle: functional properties and central actions Physiol Rev 1992 72 623 666 1626033 10.1152/physrev.1992.72.3.623

[B55] Jankowska E McCrea D Rudomin P Sykova E Observations on neuronal pathways subserving primary afferent depolarization J Neurophysiol 1981 46 506 516 7299431 10.1152/jn.1981.46.3.506

